# Causes of Pneumonia Epizootics among Bighorn Sheep, Western United States, 2008–2010

**DOI:** 10.3201/eid1803.111554

**Published:** 2012-03

**Authors:** Thomas E. Besser, Margaret A. Highland, Katherine Baker, E. Frances Cassirer, Neil J. Anderson, Jennifer M. Ramsey, Kristin Mansfield, Darren L. Bruning, Peregrine Wolff, Joshua B. Smith, Jonathan A. Jenks

**Affiliations:** Washington State University, Pullman, Washington, USA (T.E. Besser, M.A. Highland, K. Baker);; Washington Animal Disease Diagnostic Laboratory, Pullman (T.E. Besser);; US Department of Agriculture Agricultural Research Service, Pullman (M.A. Highland);; Idaho Department of Fish and Game, Lewiston, Idaho, USA (E.F. Cassirer);; Montana Fish, Wildlife and Parks, Bozeman, Montana, USA (N.J. Anderson, J.M. Ramsey);; Washington Department of Fish and Wildlife, Spokane Valley, Washington, USA (K. Mansfield);; US Department of Agriculture Animal and Plant Health Inspection Service, Olympia, Washington, USA (D.L. Bruning);; Nevada Department of Wildlife, Reno, Nevada, USA (P. Wolff);; South Dakota State University, Brookings, South Dakota, USA (J.B. Smith, J.A. Jenks)

**Keywords:** animal diseases, etiology, Mycoplasma ovipneumoniae, pulsed-field gel electrophoresis, ribosomal spacer DNA, bacteria, pneumonia, epizootic, bighorn sheep

## Abstract

Epizootic pneumonia of bighorn sheep is a devastating disease of uncertain etiology. To help clarify the etiology, we used culture and culture-independent methods to compare the prevalence of the bacterial respiratory pathogens *Mannheimia haemolytica, Bibersteinia trehalosi, Pasteurella multocida,* and *Mycoplasma ovipneumoniae* in lung tissue from 44 bighorn sheep from herds affected by 8 outbreaks in the western United States. *M. ovipneumoniae*, the only agent detected at significantly higher prevalence in animals from outbreaks (95%) than in animals from unaffected healthy populations (0%), was the most consistently detected agent and the only agent that exhibited single strain types within each outbreak. The other respiratory pathogens were frequently but inconsistently detected, as were several obligate anaerobic bacterial species, all of which might represent secondary or opportunistic infections that could contribute to disease severity. These data provide evidence that *M. ovipneumoniae* plays a primary role in the etiology of epizootic pneumonia of bighorn sheep.

## Abstract 2

*Mycoplasma ovipneumoniae* is a primary pathogen.

In North America, epizootic pneumonia is a devastating, population-limiting disease of bighorn sheep (*Ovis canadensis*) (*1**–**5*). Anecdotal and experimental evidence suggests that in at least some instances, this disease may be introduced into bighorn sheep populations by contact with domestic sheep or goats (*5**,**6*). When the disease is first introduced, outbreaks affect animals of all ages (*1**–**3*). During subsequent years or decades, sporadic cases of pneumonia in adult sheep and annual epizootics of pneumonia in lambs may continue (*7**–**10*).

Considering the dramatic and severe character of epizootic bighorn sheep pneumonia, the etiology is surprisingly unclear. Findings of gross and histopathologic examinations of lung tissue strongly suggest bacterial etiology: anterior–ventral distribution, suppurative inflammation, and abundant bacterial colonies. In domestic ruminants, bacterial pneumonia frequently occurs secondary to viral infections or other pulmonary insults, but extensive efforts to detect such underlying factors for bighorn sheep pneumonia have generally been nonproductive. For example, although evidence of infection or exposure to respiratory viruses, especially respiratory syncytial virus and parainfluenza virus, is frequently found in healthy and pneumonia-affected populations, no consistent association between the disease and any virus has been found (*11**–**13*). As a result, most research attention has been directed toward bacterial respiratory pathogens that may act as primary infectious agents, particularly leukotoxin-expressing *Mannheimia haemolytica,* which is highly lethal to bighorn sheep after experimental challenge (*5**,**14*). Other *Pasteurellaceae*, particularly *Bibersteinia trehalosi* and *Pasteurella multocida,* have been more frequently isolated from pneumonia-affected animals during natural outbreaks than has *M. haemolytica* (*11**,**12**,**15*). Another candidate pathogen, *Mycoplasma ovipneumoniae*, has recently been isolated from pneumonia-affected bighorn sheep during 2 epizootics (*11**,**16**,**17*); antibodies against this agent were detected in bighorn sheep from 9 populations undergoing pneumonia epizootics but were absent in 9 nonaffected populations (*17*). In experiments, *M. ovipneumoniae* has been shown to predispose bighorn sheep to *M. haemolytica* pneumonia (*18*). When *M. ovipneumoniae–*free domestic sheep were commingled with bighorn sheep, the bighorn sheep survived at unprecedented rates (*19*).

Development of effective methods for managing, preventing, or treating an infectious disease requires a clear understanding of its underlying etiology. However, clarifying the etiology can be difficult, particularly for primary infections (e.g., HIV) that are characteristically associated with multiple opportunistic infections that may be more lethal than the epidemic agent itself. During 2008–2010, epizootic pneumonia of bighorn sheep was detected in at least 5 western US states. These epizootics provided an opportunity to conduct a comparative study of the etiology of this disease (Table 1).

**Table 1:** Bighorn sheep populations included in study of populations affected by epizootic pneumonia, western United States, 2008–2010*

Population	Status†	Population size	% Dead or culled‡
East Fork Bitterroot, MT	Pneumonic	200–220	50
Bonner, MT	Pneumonic	160–180	68
Lower Rock Creek, MT	Pneumonic	200	43
Anaconda, MT	Pneumonic	300	50
East Humboldt/Ruby Mountains, NV	Pneumonic	160–180	80
Yakima Canyon, WA	Pneumonic	280	33
Spring Creek, SD	Pneumonic	≈40 lambs born	95 lambs
Hells Canyon, OR and WA	Pneumonic	≈170 lambs born	77 lambs
Quilomene, WA	Healthy	160	2
Asotin Creek, WA	Healthy	100	0

*MT, Montana; NV, Nevada; SD, South Dakota; WA, Washington; OR, Oregon. †Pneumonic, populations with confirmed epizootic pneumonia restricted to lambs (Spring Creek and Hells Canyon) or not age restricted (all other pneumonic populations); healthy, populations with no evidence of epizootic pneumonia. ‡Estimated percentage of the population that died or was culled during the epizootic.

Conventional microbiological methods can fail to isolate agents because of their fastidious in vitro growth requirements or intermicrobial interactions; thus, for agent isolation, we used 2 culture-independent methods (agent-specific PCRs and 16S clone libraries) in addition to conventional bacterial cultures (*17**,**20**–**22*). We expected that primary etiologic agents could be differentiated from opportunistic agents by 1) their detection at high prevalence in affected animals, 2) the presence of single (clonal) strain types within each outbreak, and 3) their uncommon or lack of detection in animals from healthy populations (*11**,**22**–**24*). Therefore, to clarify the etiology of epizootic pneumonia, we applied these criteria to the bacterial respiratory pathogens detected in multiple bighorn sheep epizootics.

## Materials and Methods

### Bighorn Sheep Populations

The study sample consisted of 8 demographically independent bighorn sheep populations in 5 states (Montana, Nevada, Washington, Oregon, and South Dakota) that had been affected by epizootic pneumonia during 2008–2010 and for which lung tissue specimens from >4 affected animals were available (Table 1). In 6 of these populations, the disease affected bighorn sheep of all ages; in the other 2 populations, in which the disease had previously affected sheep of all ages, the disease was restricted to lambs. Convenience samples were selected among those available from each epizootic: the sample of pneumonia-affected animals consisted of the first 4–6 sheep for which pneumonia had been confirmed by gross or microscopic lesions. Sheep initially selected for analysis but later determined to have lacked gross or microscopic lesions characteristic of pneumonia were retained in the study but analyzed separately. Negative controls consisted of animals with no gross or histopathologic evidence of pneumonia that died or were culled from 2 closely observed healthy populations.

### Bacteriologic Cultures

Surfaces of affected lung tissue specimens were seared, and swab samples of deeper tissues were obtained and streaked onto Columbia blood agar plates (Hardy Diagnostics, Santa Maria, CA, USA). *Pasteurellaceae* were isolated and identified by using routine methods (*25*) and then stored at −80^°^C in 30% buffered glycerol in brain–heart infusion agar (Hardy Diagnostics).

### DNA Template Preparation

DNA was extracted from 1.0–1.5 mL of fluid collected from 1–2 g of fresh-frozen lung tissue macerated in 1 mL of phosphate buffered saline for 5 min by using a stomacher (Seward Stomacher 80 Laboratory Blender, Bohemia, NY, USA). DNA was extracted by using a QIAamp mini kit (QIAGEN, Valencia, CA, USA) according to the manufacturer’s protocol.

### PCR Detection of Respiratory Pathogens and *lktA*

To detect *M. haemolytica, P. multocida, B. trehalosi*, *lktA*, and *M. ovipneumoniae*, we used previously published PCR protocols with minor modifications (Table 2). All reactions were conducted individually in 20-μL volumes containing 2 μL of DNA template (5–1,000 ng/μL), 10 μL of master mix (QIAGEN Hotstar mix for *P. multocida*, *M. ovipneumoniae*, and *lktA* and QIAGEN Multiplex PCR mix for *B. trehalosi* and *M. haemolytica*), and primers at 0.2 μmol (*P. multocida*, *M. haemolytica*, *and B. trehalosi*), 2 μmol (*M. ovipneumoniae*), or 0.5 μmol (*lktA*). Thermocycler conditions included an initial denaturation step at 95^°^C (15 min) for all agents and a final extension step at 72^°^C (5 min, except final extensions for *P. multocida* and *lktA* were 9 and 10 min, respectively). Cycling conditions used were as follows: for *M. ovipneumoniae*, 30 cycles at 95^°^C for 30 s, at 58^°^C for 30 s, and at 72^°^C for 30 s; for *B. trehalosi* and *M. haemolytica,* 35 cycles at 95^°^C for 30 s, at 55^°^C for 30 s, and at 72^°^C for 40 s; and for *P. multocida* and *lktA,* 30 cycles at 95^°^C for 60 s, at 55^°^C for 60 s, and at 72^°^C for 60 s. Amplicons were examined in UV light after electrophoresis in 1.2% agarose gel containing 0.005% ethidium bromide in 0.5× Tris/borate/EDTA buffer at 7 V/cm.

**Table 2:** PCR primers used to detect etiologic agents of pneumonia in bighorn sheep, western United States, 2008–2010

Species (gene target)	Primer	Primer sequence, 5′ → 3′	Reference
*Mannheimia haemolytica, Bibersteinia* *trehalosi*, *M. haemolytica* (*gcp*)	Mhgcp	AGAGGCCAATCTGCAAACCTCG	(*21*)
	MhgcpR	GTTCGTATTGCCCAACGCCG	(*21*)
*Bibersteinia trehalosi* (*sodA*)	BtsodAF	GCCTGCGGACAAACGTGTTG	(*21*)
	BtsodAR	TTTCAACAGAACCAAAATCACGAATG	(*21*)
Leukotoxin (*lktA*)	F	TGTGGATGCGTTTGAAGAAGG	(*26*)
	R	ACTTGCTTTGAGGTGATCCG	(*26*)
*Pasteurella multocida* (*kmt1*)	KMT1T7	ATCCGCTATTTACCCAGTGG	(*27*)
	KMT1SP6	GCTGTAAACGAACTCGCCAC	(*27*)
*Mycoplasma ovipneumoniae* (16S)	LMF	TGAACGGAATATGTTAGCTT	(*28*)
	LMR	GACTTCATCCTGCACTCTGT	(*28*)
*M. ovipneumoniae* (16S–23S intergenic spacer)	MoIGSF	GGAACACCTCCTTTCTACGG	This study
	MoIGSR	CCAAGGCATCCACCAAATAC	This study

### 16S Analyses

To detect predominant microbial populations in the pneumonic lung tissues, we used a culture-independent method (*17*). In brief, we aseptically collected two 1-g samples of lung tissue from sites at least 10 cm apart in grossly abnormal (consolidated) tissue from 16 pneumonia-affected animals, including 2 from each outbreak. Tissues were stomached and DNA was extracted (DNeasy Blood and Tissue Kit; QIAGEN) from 100-μg aliquots of each homogenate. Segments of 16S rDNA were PCR amplified and cloned. Insert DNA was sequenced (vector primers T3 and M13, BigDye version 3.1, ABI PRISM Genetic Analyzer; Applied Biosystems, Foster City, CA, USA) from 16 clones derived from each homogenate, resulting in 32 sequences from each animal. DNA sequences were assigned to species (>99% identity) or genus (>97% identity) according to BLASTN GenBank search results (*29*). Clone sequences may be accessed in GenBank under accession nos. JN857366–857894.

### Pulsed-Field Gel Electrophoresis for *Pasteurellaceae*

When available, *Pasteurellaceae* isolated from the study animals were obtained from the Washington Animal Disease Diagnostic Laboratory (Pullman, WA, USA). If such isolates were unavailable, we substituted banked isolates from other bighorn sheep involved in the same outbreaks. Isolates were subjected to pulsed-field gel electrophoresis (PFGE) performed on a CHEF-DRII PFGE apparatus (Bio-Rad, Hercules, CA, USA) in 1% agarose gel (Seakem Gold Agarose; FMC Bio Products, Rockland, MD, USA) in 0.5× Tris borate EDTA buffer at 14^°^C for 20 h at 6 V/cm and a linear ramp of 1.0–30.0 s for *ApaI* or 0.5–40.0 s for *Sma*I. *Salmonella* serovar Braenderup H9812, digested with *Xba*I for 3 h at 37^°^C, was used as a size standard on each gel. Gels were stained with ethidium bromide and photographed under UV transillumination. PFGE data were analyzed by using BioNumerics version 4.6 (www.applied-maths.com/bionumerics/bionumerics.htm) with Dice coefficients and the unpaired pair group method with arithmetic means for clustering; tolerance and optimization parameters were set at 1%.

### Intergenic Spacer Region Sequence typing for *M. ovipneumoniae*

In a preliminary study performed in our laboratory, ribosomal operon intergenic spacer (IGS) regions of *M. ovipneumoniae* from isolates from 6 bighorn sheep populations were amplified by using the method described by Kong et al. (*30*) and sequenced (Amplicon Express, Pullman, WA, USA). Sequences were aligned and clustered by using Lasergene software (DNASTAR, Inc., Madison WI, USA). Each isolate exhibited a different IGS sequence, demonstrating the utility of IGS sequences for identifying strain diversity (data not shown). We used Primer3 software (http://frodo.wi.mit.edu/primer3/) to develop primers flanking the variable IGS region, conserved among *M. ovipneumoniae* isolates, and divergent from IGS regions of the second most common sheep upper respiratory mycoplasma, *M. arginini* (Table 2). IGS PCR products were sequenced, and sequences were aligned and clustered by using Lasergene software. IGS sequences can be accessed in GenBank under accession nos. JN857895–857934.

### Statistical Analyses

To evaluate the agreement between results of bacteriologic cultures and PCR tests for detection of *P. multocida, M. haemolytica,* and *B. trehalosi*, we used Cohen κ coefficients (*31*). To assess overall differences in prevalence of specific bacterial respiratory pathogens, we used χ^2^ tests; for pairwise comparisons, we used the Marascuilo procedure (*32*) to control for multiple comparison problems, which might affect error rates. To assess associations between prevalence of different respiratory bacteria and mortality rates among different bighorn sheep populations, we used the Pearson correlation coefficient.

## Results

We detected 4 previously reported bacterial respiratory pathogens of bighorn sheep. We detected *M. haemolytica, B. trehalosi,* and *P. multocida* by using aerobic culture and species-specific PCR and *M. ovipneumoniae* by using PCR alone (*20*) (Table 3; Table A1). Agreement between detection by culture and PCR varied by agent, ranging from no agreement (*M. haemolytica*, κ −0.02), to fair agreement (*B. trehalosi*, κ 0.22), to good agreement (*P. multocida*, κ 0.76). For the purposes of the following analyses, animals for which any agent was detected by either method were considered positive for that agent. Among the targeted agents, 3 (*B. trehalosi*, *M. haemolytica*, and *M. ovipneumoniae*) were detected in >1 animals from all 8 outbreak-affected populations and 1 (*P. multocida*) was detected in animals from 5 outbreak-affected populations (Table 3).

**Table 3:** Prevalence of organisms in bighorn sheep, western United States, 2008–2010*

Population	No. tested	No. (%) detected				
		*Bibersteinia trehalosi*	*Mannheimia haemolytica*	*Pasteurella multocida*	*lktA*	*Mycoplasma ovipneumoniae*
Affected during epizootic						
East Fork Bitterroot, MT	5	3†	1	4	2	4
Anaconda, MT	5	5	5	5	0	5
Bonner, MT	6	2	2	0	0	6
Lower Rock Creek, MT	4	3	3	3	0	3
East Humboldt and Ruby Mountains, NV	6	6	5	0	1	6
Spring Creek, SD	5	5	3	4	3	5
Yakima Canyon, WA	8	5	4	5	0	8
Hells Canyon, OR and WA	5	5	2	0	4	5
Total	44	34 (77.3)	25 (56.8)	21 (47.7)	10 (22.7)	42 (95.5)
Healthy during epizootic						
Yakima Canyon, WA	6	5	3	0	1	2
Bonner, MT	1	0	0	0	0	1
Spring Creek, SD	1	1	1	0	0	0
Total	8	6 (75.0)	4 (50.0)	0	1 (12.5)	3 (37.5)
Healthy, no epizootic						
Quilomene, WA	3	1	3	0	0	0
Hells Canyon (Asotin Creek), OR and WA	2	2	1	1	0	0
Total	5	3 (60)	4 (80)	1 (20)	0	0

*Organisms detected by PCR and/or aerobic culture. MT, Montana; NV, Nevada; SD, South Dakota; WA, Washington; OR, Oregon. †Number of bighorn sheep tested in which the given agent was detected by PCR and/or bacteriologic culture.

Frequency of detecting *M. haemolytica*, *B. trehalosi*, *P. multocida*, and *M. ovipneumoniae* from pneumonia-affected animals differed significantly (χ^2^ 26.2, 3 df, p*<*0.0001). *M. ovipneumoniae* (n = 42, 95%) was detected significantly more often than any other agent except *B. trehalosi* (n = 35, 73%; Marascuilo procedure, p*<*0.05). Detection of *lktA*, a gene encoding the leukotoxin expressed by *B. trehalosi* and *M. haemolytica*, was then analyzed as a surrogate for virulent *M. haemolytica* and/or *B. trehalos* because these species, if lacking *lktA*, would be considered to have greatly reduced or no virulence (*33*). Prevalence of *lktA* (n = 10, 22.7%) was significantly lower than that of *P. multocida* (n = 21, 47.7%, Marascuilo procedure, p<0.05).

Frequency of detecting *B. trehalosi* and *P. multocida* differed significantly among outbreaks (p = 0.002 and 0.001, respectively). Similarly, PCR-based detection of *lktA* differed among outbreaks (p = 0.003). Although such differences could potentially contribute to the significant differences in disease severity and mortality rates among the epizootics in this study (χ^2^184.7, 7 df, p<0.0001), the prevalence of *B. trehalosi*, *P. multocida*, or *lktA* did not correlate with estimated mortality rates in the 8 outbreaks included in this study (Tables 1, 3).

Strain typing to evaluate the genetic similarity of bacterial pathogens within and among outbreaks (*23*) detected only single IGS types of *M. ovipneumoniae* within each outbreak, whereas distinctly different IGS types were found for each epizootic with the exception of 2 populations in Montana (Figure). In contrast, the PFGE strain types of *Pasteurellaceae* isolated from within single outbreaks ranged from 0 to 7, including 0–7 *B. trehalosi* strains and 0–2 *P. multocida* strains (Table 4; Table A1). Assessment of strain type diversity of *M. haemolytica* within outbreaks was not possible because this species was isolated only 1 time.

**Figure:**
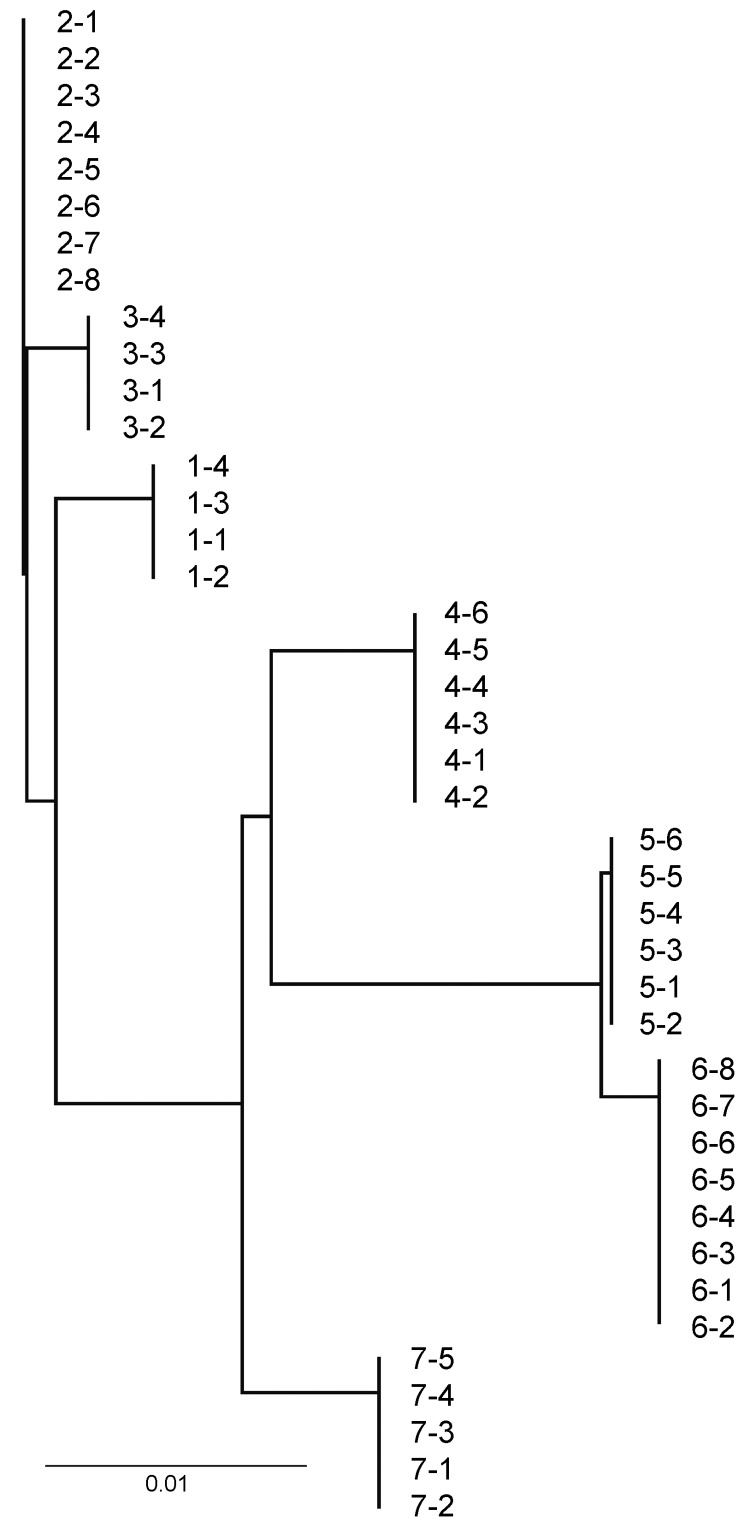
Neighbor-joining tree of ribosomal intergenic spacer region DNA sequences of *Mycoplasma ovipneumoniae* PCR-amplified from bighorn sheep lung tissues, western United States, 2008–2010. Isolate codes are those from Table 4 and Table A1. Scale bar indicates nucleotide substitutions per site.

**Table 4:** Strain types identified in lung tissue from bighorn sheep, western United States, 2008–2010*

Population	*Pasteurellaceae* (no. animals)†	*Mycoplasma ovipneumoniae *(no. animals)‡
East Fork Bitterroot, MT	Btre21, Mhae5, Pmul5 (1 each); Pmul24 (5)	Movi3 (5)
Bonner, MT	No isolate available	Movi4 (6)
Lower Rock Creek. MT	Btre1 (1); Pmul22 (3)	Movi6 (3)
Anaconda, MT	Pmul24 (5)	Movi6 (5)
East Humboldt and Ruby Mountains, NV	Btre7 (2); Btre8 (3)	Movi5 (4)
Yakima Canyon, WA	Btre8, Pmul 22 (1 each); Pmul20 (3); Btre9, 13 (5 each)	Movi2 (7)
Spring Creek, SD	Btre10, 11, 12, 17, 19 (1 each); Pmul20 (2)	Movi7 (5)
Hells Canyon, OR and WA	Btre 1, 2, 3, 4, 14, 15, 16 (1 each)	Movi1 (4)
Quilomene, WA	Mhae23 (1); Btre8 (2)	None detected
Asotin Creek, WA	No isolate available	None detected

*MT, Montana; NV, Nevada; SD, South Dakota; WA, Washington; OR, Oregon. †*Pasteurellaceae* cluster assignments based on *apa*I pulsed-field gel electrophoresis profiles with Dice coefficients clustered by unpaired pair group method with arithmetic means (threshold, 90% identity). ‡*M. ovipneumoniae* strain types identified by ribosomal 16S–23S intergenic spacer region DNA sequences.

Among the agents and genes tested, *M. ovipneumoniae* was the only agent or gene that was detected at different frequencies for animals from epizootic-affected compared with non–epizootic-affected populations (Table 3; p<0.001). The frequency of *M. ovipneumoniae* and *P. multocida* detection in non–pneumonia-affected animals culled from epizootic populations was intermediate, significantly lower than that in pneumonia-affected animals (p<0.01).

Partial 16S ribosomal DNA sequences of 527 clones, including ≈30 clones from each of 2 animals from each epizootic, detected the targeted respiratory pathogens *P. multocida*, *M. ovipneumoniae*, *B. trehalosi*, and *M. haemolytica*, although the latter accounted for <1% of the identifications. Also predominant were several obligate anaerobic bacteria (*Fusobacterium necrophorum*, *Prevotella* spp., *Clostridium* spp., and *Bacteroides* spp.) (Table 5).

**Table 5:** Bacteria detected in lung tissue from bighorn sheep with pneumonia, western United States, 2008–2010*

Bacterial species	Clone sequences, no. (%)	No. animals†	No. populations‡
*Fusobacterium* spp.	112 (21.3)	8	5
*Pasteurella multocida*	67 (12.7)	5	4
*Prevotella* spp.	57 (10.8)	9	5
*Mycoplasma ovipneumoniae*	52 (9.9)	5	4
*Bibersteinia trehalosi*	46 (8.7)	4	3
*Clostridium* spp.	42 (8.0)	10	7
*Bacteroides* spp.	16 (3.0)	7	5
*Acinetobacter* spp.	14 (2.7)	3	3
*Streptococcus* spp.	13 (2.5)	1	1
*Pseudomonas* spp.	7 (1.3)	2	2
*Eubacterium* spp.	6 (1.1)	4	3
*Pasteurellaceae* spp.	6 (1.1)	2	2
*Ruminococcus* spp.	6 (1.1)	3	3

*rDNA sequence analysis was used. Only species identifications comprising >1% of sequences are reported. †Number of animals in which the bacterial species was detected, total 16 animals (2 animals each from 8 populations). ‡Number of populations in which the bacterial species was detected, total 8 populations.

## Discussion

The results of this study support the hypothesis that *M. ovipneumoniae* is a primary agent in the etiology of epizootic bighorn sheep pneumonia in populations across the western United States and that it acts to induce secondary infection with opportunistic pathogens. *M. ovipneumoniae* was detected in the pneumonic lungs of >95% of study animals involved in the 8 discrete pneumonia epizootics, significantly more frequently than any of the other respiratory agents sought except the bighorn sheep commensal bacterium *B. trehalosi* (*34**,**35*). We identified identical ribosomal IGS strains of *M. ovipneumoniae* within the affected animals in each outbreak, consistent with epizootic spread (*24*); *M. ovipneumoniae* was not detected in the healthy populations sampled. Of note, the 2 populations in which identical IGS strains of *M. ovipneumoniae* were detected were separated by only ≈20 miles, suggesting the possibility that this strain was transmitted among these populations by movement of >1 *M. ovipneumoniae*–infected bighorn sheep.

The normal host range of *M. ovipneumoniae* (members of Old World *Caprinae*, including domestic sheep and mouflon, a closely related Eurasian sheep species) is consistent with many observations that epizootic bighorn sheep pneumonia frequently follows contact with these hosts (*5**,**19*). Previous experiments in which bighorn sheep were commingled with domestic sheep or mouflon each resulted in epizootic bighorn sheep pneumonia and, cumulatively, the death of 88 (98%) of 90 bighorn sheep; similar commingling experiments with other ungulates (deer, elk, llamas, horses, cattle, goats, mountain goats) resulted at most in sporadic deaths from bighorn sheep pneumonia (4 [7%] of 56) (*19*). In a recent study in which bighorn sheep were commingled with *M. ovipneumoniae*–free domestic sheep, the lack of epizootic bighorn sheep pneumonia was unprecedented (*19*). Together, these data support the hypothesis that bighorn sheep epizootic pneumonia results from cross-species transmission of *M. ovipneumoniae* from its normal host(s) to a naive, highly susceptible host: bighorn sheep.

Each of the other specific potential respiratory pathogens targeted failed to fulfill >1 expectations for a primary etiologic agent. *B. trehalosi* was detected in most animals regardless of their health status; exhibited diverse strain types within epizootics; and in most instances was detected in the absence of *lktA*, consistent with the nontoxigenic strains widely distributed in healthy and pneumonic bighorn sheep (*36*). *M. haemolytica* was similarly detected in about half of the animals regardless of their health status and in the absence of *lktA*. *P. multocida* was not detected at all in animals involved in 3 of the epizootics, but in those outbreaks in which it was present, it was detected at high prevalence and somewhat more frequently in pneumonia-affected than in healthy bighorn sheep. Furthermore, multiple isolates from those epizootics in which it was detected shared a high degree of genetic similarity, consistent with epizootic transmission (*24*).

The frequencies with which *B. trehalosi*, *P. multocida*, and *lktA* were detected from animals in the different epizootics differed significantly, although this finding did not correlate with mortality rates (Table 1). This conclusion is limited, however, by the possible confounding effect of the extensive culling conducted in several areas of the epizootics examined in this study. More research into factors that affect the severity of bighorn sheep pneumonia epizootics is clearly needed.

The analysis of prevalence of bacterial respiratory pathogens in the lung tissues of healthy animals from unaffected populations was comparatively limited by the small number of control specimens available. To more clearly define the prevalence, infectivity, and virulence of *M. ovipneumoniae*, sampling of additional healthy bighorn sheep populations is needed. Although *M. ovipneumoniae* was not detected in the negative control animals examined in this study and although serologic evidence of exposure to *M. ovipneumoniae* is uncommon or rare in healthy bighorn sheep populations (*17*), several apparently healthy bighorn sheep populations with serologic and/or PCR evidence of exposure to *M. ovipneumoniae* have been identified (data not shown). This finding demonstrates that not all exposures to this agent result in epizootic bronchopneumonia or, perhaps, that unrecognized previous epizootics had occurred. To clarify these findings, more research, specifically including longitudinal observational studies and investigation of strain differences in virulence of *M. ovipneumoniae* (*37**,**38*), is needed.

Our universal eubacterial 16S rDNA approach used analysis of small clone libraries from each animal to detect those agents representing >10% of the 16S operons in lung tissue with high (>95%) confidence. The 3 most frequently detected aerobic bacterial agents detected by using this method were *P. multocida*, *M. ovipneumoniae*, and *B. trehalosi*; detection of *M. haemolytica* was rare (0.19%) despite the high frequency of its detection by the more sensitive PCR. This finding is surprising because *M. haemolytica* has been regarded as the principal pneumonia pathogen of bighorn sheep (*5*). It has been argued that because *B. trehalosi* inhibits or kills *M. haemolytica* in coculture, the same effect in vivo may block detection of *M. haemolytica* in bighorn sheep lungs (*21*), but this argument cannot explain the dearth of *M. haemolytica* detected in this study because the proportion of lungs that were positive for *M. haemolytica* by PCR was actually lower in the absence of *B. trehalosi* (Table A1). Furthermore, the lung tissues from animals affected by the 5 epizootics in Washington or Montana were obtained from bighorn sheep that were coughing and culled in an attempt to prevent further transmission of the disease; therefore, these specimens could represent animals at earlier stages of the disease when more consistent presence of causal agents would be expected. 

Consistent with previous reports of bighorn sheep in Hells Canyon (*17*), the predominance of obligate anaerobes (*Fusobacterium*, *Prevotella*, *Clostridium*, and *Bacteroides* spp.) among the lung flora was consistent with decreased clearance of inhaled oral flora from the lower respiratory tract. Impaired clearance of inhaled flora is expected subsequent to infection by virulent *M. ovipneumoniae* (*38*) or by leukotoxin-expressing *Pasteurellaceae* (*39*), albeit by different mechanisms.

To our knowledge, only 1 other study of epizootic bighorn sheep pneumonia has reported comparative microbiological findings from pneumonia-affected animals involved in multiple discrete epizootics. Aune et al. (*12*) reported that *Pasteurellaceae* cultured from pneumonia-affected animals differed somewhat among 4 bighorn sheep pneumonia epizootics in Montana during 1991–1996. *P. multocida* was isolated from pneumonic lung tissues of >1 animals during all 4 epizootics, although prevalence exceeded 50% during only 1 epizootic. *Pasteurellaceae* biotypes corresponding to *B. trehalosi* were isolated from animals involved in 3 of the 4 outbreaks, and *Pasteurellaceae* biotypes corresponding to *M. haemolytica* were isolated from animals in only 1 outbreak. The microbiology of epizootic pneumonia in Hells Canyon also has been described (*11**,**15**,**40*); results were broadly comparable to the conventional microbiology results reported here for *Pasteurellaceae*. All these studies differed from the study reported here in that the conventional microbiological methods used failed to recognize *M. ovipneumoniae* in affected lung tissues.

In summary, of the bacterial respiratory pathogens evaluated, *M. ovipneumoniae* was the only agent for which the data consistently met the criteria for a primary etiologic agent across all outbreaks. In contrast, the data were inconsistent with regard to a primary etiologic role for any *Pasteurellaceae* species. The likelihood of *M. ovipneumoniae* having a primary role in bighorn sheep pneumonia is consistent with the association between some epizootics of this disease and contact with domestic sheep because the latter carry this agent at high prevalence. Identification of *M. ovipneumoniae* as the epizootic agent of bighorn sheep pneumonia may provide a useful focus for the development of specific preventative or therapeutic interventions.

## Appendix

**Table A1:** PCR and aerobic culture results for individual bighorn sheep, western United States, 2008–2010*

Health status	Population	Sheep ID	*Mannheimia haemolytica*			*Bibersteinia trehalosi*			*lktA*		*Pasteurella multocida*			*Mycoplasma ovipneumoniae*	
			Culture	PCR		Culture	PCR		PCR		Culture	PCR		PCR	Isolate no.
PP	Hells Canyon, OR and WA	10WA02	0	0		0	1		1		0	0		1	
PP	Hells Canyon, OR and WA	10OR09	NT	1		NT	1		1		NT	0		1	1-4
PP	Hells Canyon, OR and WA	10OR05	0	0		1	1		0		0	0		1	1-1
PP	Hells Canyon, OR and WA	10OR06	0	0		1	1		1		0	0		1	1-3
PP	Hells Canyon, OR and WA	10OR07	0	1		1	1		1		0	0		1	1-2
PP	East Fork Bitterroot, MT	185887	1	1		1	1		1		0	0		1	3-2
PP	East Fork Bitterroot, MT	185888	0	0		0	0		0		1	1		1	3-1
PP	East Fork Bitterroot, MT	185960	0	0		0	0		0		1	1		1	3-3
PP	East Fork Bitterroot, MT	185976	0	0		0	1		0		1	1		0	NA
PP	East Fork Bitterroot, MT	185979	0	0		0	1		1		1	1		1	3-4
PP	Anaconda, MT	186759	0	1		0	1		0		1	1		1	6-2
PP	Anaconda, MT	186760	0	1		0	1		0		1	1		1	6-1
PP	Anaconda, MT	186761	0	1		0	1		0		1	1		1	6-3
PP	Anaconda, MT	186762	0	1		0	1		0		1	1		1	6-5
PP	Anaconda, MT	186764	0	1		0	1		0		1	1		1	6-4
PP	Bonner, MT	186054	0	0		0	1		0		0	0		1	4-3
PP	Bonner, MT	186088	0	0		0	0		0		0	0		1	4-4
PP	Bonner, MT	186091	0	0		0	0		0		0	0		1	4-5
PP	Bonner, MT	186092	0	1		0	0		0		0	0		1	5-6
PP	Bonner, MT	186093	0	1		0	0		0		0	0		1	4-1
PP	Bonner, MT	186109	0	0		0	1		0		0	0		1	4-2
PP	Lower Rock Creerk, MT	186249	0	0		0	0		0		1	1		1	6-7
PP	Lower Rock Creerk, MT	186139	0	1		1	1		0		1	1		1	6-6
PP	Lower Rock Creerk, MT	186195	0	1		0	0		0		0	0		0	NA
PP	Lower Rock Creerk, MT	186198	0	1		0	1		0		1	0		1	6-8
PP	East Humboldt/Ruby Mountains, NV	10-3422 #28	0	0		1	1		0		0	0		1	5-3
PP	East Humboldt/Ruby Mountains, NV	WHITE RAM	0	1		1	1		1		0	0		1	5-4
PP	East Humboldt/Ruby Mountains, NV	10-2819	0	1		1	0		0		0	0		1	5-1
PP	East Humboldt/Ruby Mountains, NV	10-2860 Lamoile lamb	0	1		1	1		0		0	0		1	5-2
PP	East Humboldt/Ruby Mountains, NV	10-3273 (#19)	0	1		1	1		0		0	0		1	5-5
PP	East Humboldt/Ruby Mountains, NV	10-2927	0	1		1	1		0		0	0		1	5-6
PP	Spring Creek, SD	1144B	0	1		0	1		1		0	1		1	7-1
PP	Spring Creek, SD	1213	0	0		0	1		0		1	1		1	7-2
PP	Spring Creek, SD	912	0	1		1	1		1		0	0		1	7-3
PP	Spring Creek, SD	1123	0	0		0	1		0		1	1		1	7-4
PP	Spring Creek, SD	1152	NT	1		NT	1		1		NT	1		1	7-5
PP	Yakima, WA	DSURNA	0	1		0	1		0		1	1		1	2-2
PP	Yakima, WA	DSURNB	0	0		0	0		0		1	1		1	2-4
PP	Yakima, WA	KGURNA	0	1		0	1		0		0	1		1	2-3
PP	Yakima, WA	ZBSBNB	0	0		0	0		0		0	0		1	2-7
PP	Yakima, WA	UM2	0	1		0	1		0		0	0		1	2-7
PP	Yakima, WA	HTCSBN	0	1		0	1		0		1	1		1	2-5
PP	Yakima, WA	HTQSBN	0	0		1	1		0		0	1		1	2-6
PP	Yakima, WA	MPURNA	0	0		0	0		0		0	0		1	2-1
HH	Asotin Creek, WA	06AS25	0	1		0	1		0		0	0		0	NA
HH	Asotin Creek, WA	10WA05	0	0		1	1		0		1	0		0	NA
HH	Quilomene, WA	Q1	1	0		0	0		0		0	0		0	NA
HH	Quilomene, WA	Q2	0	1		0	0		0		0	0		0	NA
HH	Quilomene, WA	Q3	0	1		1	1		0		0	0		0	NA
HP	Bonner, MT	186112	0	0		0	0		0		0	0		1	NA
HP	Yakima, WA	DSURSF	0	0		1	0		0		0	0		0	NA
HP	Yakima, WA	JCURND	0	0		0	0		0		0	0		0	NA
HP	Yakima, WA	DSSBCP	0	1		0	1		0		0	0		0	NA
HP	Yakima, WA	SB1	0	1		1	1		0		0	0		0	NA
HP	Yakima, WA	UM1	0	1		1	1		0		0	0		1	NA
HP	Yakima, WA	HRCURS	0	0		0	1		1		0	0		1	NA
HP	Spring Creek, SD	216	0	1		1	1		0		0	0		0	NA

*PP, pneumonia-affected animal from epizootic population; OR, Oregon; WA, Washington; MT, Montana; NA, not applicable; NV, Nevada; SD, South Dakota; HP, healthy animal from epizootic population; HH, unaffected animal from non-epizootic population; 0, not detected; 1, detected; NT, not tested because of *Proteus* overgrowth. †See Figure.
